# Supplementary material for: High resolution cryo-EM structure of the helical RNA-bound Hantaan virus nucleocapsid reveals its assembly mechanisms
Source: eLife. 2019 Jan 14;8:e43075. doi: 10.7554/eLife.43075 (PMC6365055; doi:10.7554/eLife.43075)
Supplement: Figure 1—source data 1. [file elife-43075-fig1-data1.docx]

**Figure 1 – Source Data 1:** Cryo-EM data collection, refinement and validation statistics

| **Data Collection and processing** |  |
| --- | --- |
| Microscope | FEI Titan Krios |
| Camera | Gatan K2 Summit + GIF |
| Magnification | 46860 |
| Voltage (kV) | 300 |
| Number of frames | 28 |
| Electron exposure total (e^-^/A^2^) | 40 |
| Defocus range (μm) | 0.8-3.5 |
| Pixel size (Å) | 1.067 |
| Symmetry imposed | C1, helical twist -99.95°, helical rise 18.87 Å |
| Initial/Final micrographs (no.) | 4328 |
| Initial particles segments (no.) | 168,709 |
| Final particles segments (no.) | 105,665 |
| Map resolution (Å) 0.143 FSC threshold | 3.3 |
| Map resolution range (Å) | 3.2-4 |
| **Refinement** |  |
| Initial model used | 5FSG |
| Model resolution (Å) 0.5 FSC threshold | 3.4 |
| Map sharpening B factor (Å^2^) | -103 |
| Model composition (monomer) |  |
| Protein residues | 2650 |
| Ligands | 60 |
| B-factor (Å^2^) |  |
| Protein | \| 52.02 (36.64-82.38) \| \| --- \| |
| Ligand | 68.18 (62.93-73.14) |
| R.m.s deviations |  |
| Bond lengths (Å) | 0.007 |
| Bond angles (°) | 0.951 |
| **Validation** |  |
| MolProbity score | 1.48 |
| Clashscore | 2.89 |
| Poor rotamers (%) | 0.0 |
| Ramachandran plot |  |
| Favored (%) | 93.98 |
| Allowed (%) | 6.02 |
| Disallowed (%) | 0 |
